# Change of Rin1 and Stathmin in the Animal Model of Traumatic Stresses

**DOI:** 10.3389/fnbeh.2017.00062

**Published:** 2017-04-26

**Authors:** Fang Han, Jingzhi Jiang, Jinlan Ding, Hong Liu, Bing Xiao, Yuxiu Shi

**Affiliations:** Post-Traumatic Stress Disorder (PTSD) Laboratory, Department of Histology and Embryology, Basic Medical College, China Medical UniversityShenyang, China

**Keywords:** Rin1, stathmin, fear memory, single prolonged stress, post-traumatic disorder, traumatic stress

## Abstract

The molecular mechanism of fear memory is poorly understood. Therefore, the pathogenesis of post-traumatic stress disorder (PTSD), whose symptom presentation can enhance fear memory, remains largely unclear. Recent studies with knockout animals have reported that Rin1 and stathmin regulate fear memory. Rin1 inhibits acquisition and promotes memory extinction, whereas stathmin regulates innate and basal fear. The aim of our study was to examine changes in the expression of Rin1 and stathmin in different animal models of stress, particluarly traumatic stress. We used three animal traumatic stresses: single prolonged stress (SPS, which is a rodent model of PTSD), an immobilization-stress (IM) and a Loud sound stress (LSS), to examine the change and uniqueness in Rin1/stathmin expression. Behavioral tests of SPS rats demonstrated increased anxiety and contextual fear-conditioning. They showed decreased long-term potentiation (LTP), as well as decreased stathmin and increased Rin1 expression in the hippocampus and the amygdala. Expression of the stathmin effector, tubulin, and downstream molecules Rin1, Rab5, and Abl, appeared to increase. Rin1 and EphA4 were endogenously coexpressed in primary neurons after SPS stimulation. IM rats exhibited increased anxiety behavior and enhanced fear-conditioning to contextual and auditory stimuli. Similar changes in expression of Rin1/stathmin were observed in IM rats whereas no changes were observed in rats exposed to a loud sound. These data suggest that changes in expression of the Rin1 and stathmin genes may be involved in rodents with SPS and IM stresses, which provide valuable insight into fear memories under abnormal conditions, particularly in PTSD.

## Introduction

Stathmin, which is also called oncoprotein 18, is a neuronal growth-associated protein that is highly expressed in the lateral nucleus of the amygdala and related thalamic and cortical structures (Shumyatsky et al., 2002). Furthermore, it is reported to stathmin plays an important role in regulating formation/disassembly of cellular microtubules (MTs) and synaptic plasticity (Belmont and Mitchison, 1996). Rin1 is a Ras effector protein that is strongly expressed in telencephalic regions, including the cortex, hippocampus, amygdala, and striatum. Rin1 protein is normally localized in neuronal cell bodies and dendrites (Deininger et al., 2008). Rin1 showed low expression at the postnatal mouse brain, suggesting Rin1 may be dispensable for early brain but implicated Rin1 in mature neurons (Dhaka et al., 2003). A number of studies have reported high expression of both genes in cancers such as cervical, prostate, and lung cancers (Shan et al., 2012; Biaoxue et al., 2016).

An increasing number of studies have reported that stathmin plays a key role in learning and innate fear, particularly basal fear (Peschanski et al., 1993; Shumyatsky et al., 2002; Brocke et al., 2009). Stathmin knockout mice show decreased memory during amygdala-dependent fear conditioning, fail to recognize the danger in innate fear-aversive environments, and have deficits in long-term potentiation (LTP; Shumyatsky et al., 2002). Stathmin interacts with tubulin and forms heterodimers, which prevent the formation of MTs (Curmi et al., 1997). Tubulin released from the heterodimer enhances formation of MTs after phosphorylation. As a regulator of synaptic plasticity, stathmin is involved in the formation and disassembly of cellular MTs and functions (Belmont and Mitchison, 1996).

Behavioral studies of Rin1−/− mice have demonstrated enhanced learning of conditioned fear, enhanced acquisition of aversive memories, and elevated amygdalar LTP (Deininger et al., 2008; Bliss et al., 2010; Dzudzor et al., 2010), suggesting a critical role for Rin1 in acquisition and persistence of fear conditioning. Rin1−/− and stathmin −/− knockout mice both develop normally and have no alterations in spatial-dependent memory. Two downstream effectors of Rin1 signaling, Abl (Hu et al., 2005) and Rab5 (Tall et al., 2001), regulate cytoskeletal remodeling and endocytosis, respectively. Abl is activated by Rin1 and may contribute to cytoskeletal remodeling of postsynaptic dendritic spines and modulate short-term synaptic plasticity in the hippocampus (Koleske et al., 1998; Brown et al., 2005). Activated Rab5 participates in regulating endocytosis of cell surface receptors in multiple forms of Long-term depression (LTD). Rin1 also contributes to endocytosis of EphA4 in amygdalar neurons (Deininger et al., 2008).

Therefore, both genes are essential in regulating fear memory but not spatial memory. Most genes, such as the N-methyl-D-aspartate receptor, protein kinase C and calmodulin-dependent protein kinase II, are now considered to be involved in regulating multiple learning-memory pathways, including fear- and spatial-dependent memory (Goosens et al., 2000; Humeau et al., 2003; Rodrigues et al., 2004; Fourcaudot et al., 2009). However, only a few genes, including stathmin and Rin1, have been reported to be responsible for fear memory. According to the different expression in behavioral test and LTP in the knockout animals, it is possible that stathmin and Rin1 have different effects or opposite effects on the fear memory.

Post-traumatic stress disorder (PTSD) is a cognitive and emotional disorder that develops after an individual is exposed to a stressful or traumatic event such as violence or an earthquake. PTSD is characterized by re-experiencing the trauma, avoidance, negative changes in cognition/mood, and altered arousal (Adami et al., 2006; Liberzon and Martis, 2006; American Psychiatric Association, 2013). Patients with PTSD may show abnormal consolidation and retrieval of traumatic memories, causing traumatic flashbacks. Normally, traumatic memories are more robust as compared to non-traumatic memories. The hippocampus and amygdala are involved in the fear memory circuit and playing key roles in regulating fear-related emotions and schemas (Rauch and Shin, 1997; Hull, 2002; Shin et al., 2005; Hughes and Shin, 2011). Magnetic resonance imaging (MRI) studies have revealed significant reductions in hippocampal and amygdala volume in adult patients with PTSD. Functional MRI studies have determined that the amygdala is highly activated in patients with PTSD, which is not the case in the hippocampus decreases (Shin et al., 2005; Etkin and Wager, 2007; Morey et al., 2009; Murrough et al., 2011; Xiong et al., 2013). In the present study, we used the single prolonged stress (SPS), which has been widely used as PTSD animal model (Liberzon et al., 1999). During SPS, animals are restrained for 2 h, forced swim for 20-min in 20–24°C water, and finally exposed to ether anesthesia. Neuroendocrinological and behavioral evidences support that SPS rats may be more appropriate and more practical as models of fear-related human condition labeled clinically PTSD. Our previous studies with SPS model found high apoptosis ratios in neurons of both structures (Ding et al., 2010; Li et al., 2010; Han et al., 2013, 2014). Abnormal structure and function of the amygdala and hippocampus may cause abnormal memory-related symptoms.

In the present study, we used the SPS (an animal model that mimics the features of the human condition known as PTSD), an immobilization-stress (IM; a traumatic-like stress) and a Loud sound stress (LSS) to examine the change of Rin1 and stathmin in three different stresses. The changes in expression of the Rin1 and stathmin genes may be involved in traumatic-like stress model, which may be useful in traumatized human populations as well and may provide valuable insight into fear memories that are processed under abnormal conditions.

## Materials and Methods

### Animal Model Preparation and Grouping

#### Single Prolonged Stress (SPS) Experiment

A total of 80 male Wistar rats (220–250 g) were randomly divided into two groups (40 rats per group): a control group and SPS groups examined on day 7. The control rats remained in their home cages with no handling for 7 days and were killed at the same time as the SPS groups. The SPS rats underwent the SPS procedure on the first day. The SPS procedure was carried out according to the following protocol (Liberzon et al., 1999): a 2 h immobilization (compression with plastic bags), a 20 min forced swim (25°C), a 15 min rest, followed by ether anesthesia (until loss of consciousness). After SPS, the rats were *ad libitum*.

#### Immobilization-Stressed (IM) Experiment

A total of 45 male Wistar rats (220–250 g) were randomly divided into three groups (15 rats/group): a control group, a restrict stress group (IM) examined on day 1 and a restrained group examined on day 7. The rats underwent the immobilization stress procedure on the first day and underwent 1 h immobilization (compression with plastic bag). The control rats remained in their home cages with no handling.

#### Loud Sound Stress (LSS) Experiment

A total of 10 male Wistar rats (220–250 g) were randomly divided into two groups (five rats per group): a control group, LSS group (LSS) examined on day 7. The rats were placed in the chamber (23 × 23 × 35 cm) for 5 min before exposure to the loudspeaker stimulus. A sonic wave (1 min, 1000 Hz, 75 Db) was delivered through the loudspeaker. The control group was placed in the same chamber for 5 min, but without the loudspeaker stimulus.

All experimental animals were maintained as a group on a 12:12 h light/dark cycle, 19–21°C room temperature. The animals had access to food and water. All experimental procedures were approved by the ethics committee of China Medical University and conducted in accordance with the Guidelines Principles on Animal Experimentations for Laboratory Animal Science, China Medical University.

### Behavioral Test

For the freezing behavior test, 40 rats per test (20 rats per group) from SPS experiment were included; for open field (OF) test and elevated plus maze (EPM) test, the remaining 20 rats were used. The EPM test was occurred 1 day after OF test. Fifteen rats (five rats per group) from IM experiment underwent the OF test and elevated plus maze (EPM) test. The remaining 20 rats from IM model were used in the fear conditioning test.

#### Open Field (OF) Test

The open-field test was used to study anxiety-related behavior. The procedure was done as described in Han et al. (2014). The apparatus was surrounded by black walls 40 cm in height, and the floor (100 cm × 100 cm) was divided into 25 squares (20 cm × 20 cm each). During the experiment, each rat was put in the center of the OF (50 cm × 50 cm), and behavior was recorded for 5 min by an automatic analyzing system (Smart 3.0, Panlab, Barcelona, Spain). Time of center cross, the distance of center cross and total cross, and the number of rearing were recorded. The apparatus was cleaned with 70% ethanol using a wet sponge and a paper towel before the introduction of each rat. The percentage of border/center distance (distance into the border (center)/total distance), and the percentage of time in the border/center (time in the border (center)/total time) were calculated.

#### Elevated Plus Maze (EPM) Test

The procedure was done in Han et al. (2014). The EPM apparatus consists of a plus-shaped maze elevated above the floor with two oppositely positioned closed arms (50 × 10 cm), two oppositely positioned open arms (50 × 10 cm), and a center area (10 × 10 cm). At the beginning, rats were placed in the central area of the maze, facing an enclosed arm. Behavior was recorded with a video camera during the initial 5 min (Smart 3.0, Panlab, Barcelona, Spain). The apparatus was cleaned with 70% ethanol using a wet sponge before the next observation. The number of entries, the time spent and distance into open arms and into closed arms were measured. The percentage of open /closed arm time (time in the open arms (closed arms)/the time in both arms), the percentage of open /closed arm distance (distance in the open arms (closed arms)/the distance in both arms) and open arm entries (number of entries into the open arm/total number of entries in both arms) were calculated. The measures of anxiety are the percentage (%) of open arm entries and the percentage (%) of time spent on the open arms.

#### Fear Conditioning

In the fear conditioning test, 40 rats (20 rats per group) from the SPS experiment and 20 rats from IM experiment were trained, after training, rats from each experiment were separated into two groups, one for the contextual fear test and the other group for the auditory cued fear test. The procedure was referenced in Bliss et al. (2010). Meanwhile, sensibility test to the foot-shock was done.

#### Sensibility Test to the Foot-Shock

The rats were placed in the conditioning charmber (23 × 23 × 35 cm) for 3 min. After 3 min, an electrical foot shock stimulation were delivered. Current strength started from 0.05 mA and progressive increase with 0.05 mA. The minimum current strength was recorded when rats appeared the following three behavioral responses: Notice (head toward the reaction), Flinch (hint foot lift from electric shock rod) and Vocalize.

#### Contextual Fear Conditioning

The rats were placed in the conditioning chamber and the rats allowed to freely explore for 5 min. The degree of freezing during 5 min was considered as baseline freezing. After 5 min of exploration, an auditory cue (1000 Hz, 75 dB, conditioned stimulus (CS)) was presented for 30 s and an electrical foot shock (2 s 1.5 mA, unconditioned stimulus (US)) stimulation were delivered continuously during the last 2 s of the auditory cue. The presentation of CS-US repeats three times per session with 90 s interval during each repeat. Following the final footshock, the rats were returned to home-cage. Forty-eight hours after training, the rats were placed in the chamber which rats were trained and tested for freezing to the contextual fear conditioning. After 5 min, the rat was returned to home cage, the chamber was cleaned and the next phase of the experiments was started.

#### Auditory Cued Fear Conditioning

Forty-eight hours after training (the training was described in “contextual fear conditioning”), rats from a separate group were placed in a novel chamber and tested for freezing to the tone. After 2 min habituation period (pre-CS), the freezing time was measured immediately after the tone stimulation (post-CS, without foot shock) within 120 s.

For the fear conditioning test, the freezing activity was recorded and measured using Packwin 2.0 software (Panlab, Barcelona, Spain). Freezing time were used as an index of fear conditioning. Freezing was defined as immobility, excluding respiratory movements with a freezing posture. Rats remained still, sluggish, curled or crouched whilst breathing, and had a slight rocking motion.

### Long-Term Potentiation (LTP)

The rats (10 rats per group) were deeply anaesthetised by 20% urethane administered i.p. (6.5 ml/kg). The rats were positioned in a stereotaxic instrument (Harvard apparatus, Holliston, MA, USA), and the scalp was cut and retracted to expose the skull. According to the brain stereotaxic atlas of Paxinos and Watson (1998), insert the stimulating electrode with interelectrode distance of 0.4 mm into the hippocampal CA3 area (coordinates: AP 3.8 mm, ML 3.8 mm, Depth 3.8 mm) and fixed with dental cement. Then insert glass microelectrode (tip 1~2 microns in diameter, impedance 5~20 mΩ, filled with 3 mol/L KCl) into the CA1 area (coordinates: AP 3.8 mm, ML 1.8 mm, Depth 2 mm). For the amygdala, insert the stimulating electrode with interelectrode distance of 0.4 mm from the entorhinal cortex area (coordinates: AP 4.8 mm, ML 6.5 mm, Depth 9 mm) and insert glass microelectrode (tip 1~2 microns in diameter, impedance 5~20 mΩ, filled with 3 mol/L KCl) into basolateral nucleus of the amygdala (coordinates: AP 1.8 mm, ML 4.5 mm, Depth 8–8.5 mm; Yaniv et al., 2003).

First rats were given a single wave pulse stimulation (7.5 V, 0.1 ms). Each response amplitude were recorded after evoked population spike (PS). The average amplitude of six times evoked PS (1 time each 5 min) were considered as the baseline value (100%). Then single plus stimulation-induced change in PS amplitude and lasting time were recorded after giving the high frequency stimulation with 100 Hz for 5 s (high frequency stimulant, HFS). The change in more than 30% in average amplitude and maintain more than 30 min were defined as LTP and LTD. Higher were LTP; Lower were LTD.

### Western Blotting Analysis

Rats (*n* = 4 per group) without fear conditioning training were decapitated, and the brains were immediately removed and quick frozen in liquid nitrogen and stored at −80°C. The amygdala and the whole hippocampus were then dissected from brain tissue according to the atlas using a stereomicroscope. The amygdala and the hippocampus of each rat was homogenized with a buffer containing 200 mM TBS, 4% SDS, 20% glycerol, and 10% 2-mercaptoethanol, and were denatured by boiling for 5 min. Samples (50 μg/lane) were loaded on a 7.5% SDS-polyacrylamide gel, and electro-blotted onto a PVDF membrane (Millipore Corp., Bedford, MA, USA) from the gel by a semi-dry blotting apparatus (Bio-Rad Laboratories, Inc, Hercules, CA, USA). The PVDF membrane was treated with 1.5% skim milk, 0.05% Tween-20 in TBS (TBST) at 4°C overnight, and then incubated with primary antibodies (primary antibodies list were shown in Table 1) at 4°C for 24 h. After being washed three times with TBST, the blots were incubated with a second antibody (anti-mouse or anti-rabbit or anti-goat IgG-HRP from Santa Cruz; 1:1000) for 2 h at room temperature. After incubation, blots were washed three times with TBST, and then were visualized using enhanced chemiluminescence (ECL; Amersham Pharmacia Biotech, NJ, USA). The same blots were incubated with antibodies against GAPDH as positive control. The protein levels were evaluated by calculating the OD ratio. The OD of proteins and GAPDH were analyzed on the Gel Image Analysis System (Tanon 2500R, Shanghai, China). The procedures were repeated four times per rat and then calculation of four rats per group to obtain the average value of each group.

**Table 1:** **The following antibodies were used for western blotting and immunofluorescence**.

Antibody name	Company	Concentration
Goat polyclonal antibody against stathmin	Santa Cruz, USA	1:500
Mouse monoclonal antibody against tubulin	Boster, China	1:200
Mouse monoclonal antibody against Rab 5	Boster, China	1:1000
Rabbit polyclonal antibody against Rin1	Santa Cruz, USA	1:1000
Mouse monoclonal antibody against EphA4	Boster, China	1:200
Rabbit monoclonal antibody against Abl	Boster, China	1:200
Mouse monoclonal antibody against NeuN	Abcam, USA	1:500
Mouse monoclonal antibody against GAPDH	Boster, China	1:500
Mouse monoclonal antibody against GFAP	Santa Cruz, USA	1:1000

### The Immunofluorescence Experiment

Rats (four rats per group) were anesthetized with 50 mg/kg body weight sodium pentobarbital and were perfused through the heart with 4% paraformaldehyde in phosphate buffer. The brains were removed from the skull and fixed in the same fixative solution for 24 h. The brains were immersed in 30% sucrose in 0.1 M PB for 3 days for cryosections. The brains were then quickly frozen using powdered dry ice and cut into 25 μm thick frontal sections on a cryostat (Leica CM 3050, Germany). The sections were stored at 4°C before immunofluorescence. The sections were treated with 2% BSA in 0.3% Triton X-100 in PBS for 2 h at RT to block nonspecific reaction. The sections were incubated with primary antibodies (see Table 1) overnight at 4°C. For single labeling immunofluorescence, sections were incubated with a primary antibody (Stathmin, Rin1). For double labeling immunofluorescence, sections were incubated with a mixture of two antibodies (Stathmin and NeuN; Stathmin and GFAP; Rin1 and NeuN; Rin1 and GFAP; Rin1 and EphA4). After three washes with phosphate-buffered saline (PBS), sections were incubated at 37°C for 30 min with secondary antibodies. Sections were incubated with DAPI and then washed four more times with PBS and CA1 subregion was observed and the amygdala under a confocal laser scanning microscope (TI-PS100W, Nikon, Japan).

### Statistical Analyses

The results were expressed as mean ± SEM. The differences between control group and SPS groups were analyzed by student’s *T*-test after a normality test (*P* > 0.05) using SPSS 13.0 software (Armonk, NY, USA). For the IM experiment, the differences among three experimental groups were analyzed by one-way ANOVA. A level of *P* < 0.05 was considered to be statistically significant.

## Results

### Behavioral Changes in Single Prolonged Stress (SPS) Rats

#### SPS Rats Displayed Enhanced Anxiety Behavior

**Figure 1:**
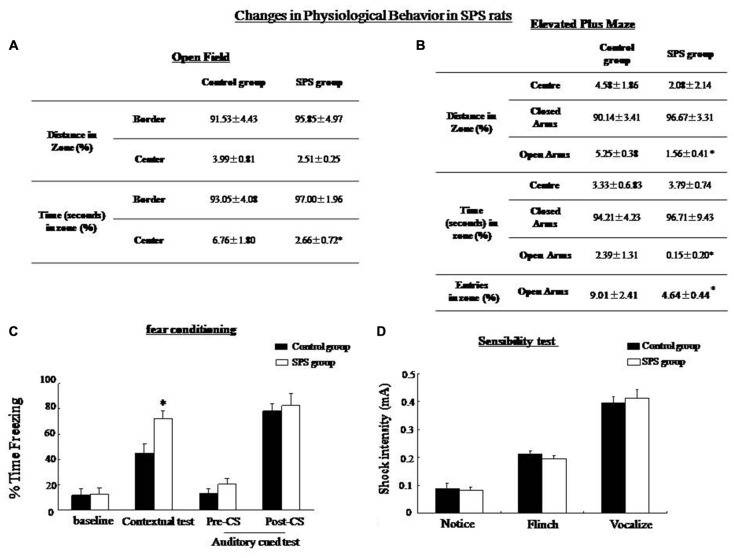
**Single prolonged stress (SPS) rats showed decreased exploratory behavior in an open field (OF) test and an elevated plus maze. (A)** OF test: SPS rats (*n* = 10) spent less time in the center zone and showed less rearing compared with the control group (*n* = 10). **(B)** Elevated plus maze: SPS rats spend shorter distances and spent less number/time in the open arms compared with control rats. **(C)** Conditioning test: the percentage of time spent freezing in contextual fear conditioning was significantly higher in the SPS rats than in the control rats (*P < 0.05 vs. the control group), but no difference in auditory cue memory between control and SPS groups. **(D)** Sensibility test to the foot-shock, no significant difference in minimum current strength which induced notice, flinch and vocalize was found between control and SPS rats.

OF test and elevated plus maze (EPM) were utilized to measure anxiety level, exploratory activity, and aversion. In the OF test, the rats were placed in a novel environment and they naturally avoided the open space in the center. The results of the OF test showed a significant decrease in time in the center of rats after exposure to SPS compared with control rats (student’s *T*-test, *n* = 10, *P* < 0.05), which is related to increased anxiety (Figure 1A). The EPM was a conflict test between avoiding the open arms of a maze and exploring a new area. The results showed no significant differences in time or distance (student’s *T*-test, *n* = 10, *P* = 0.053) in the closed arms. Significantly decreased distance, time and numbers of entries in open arms (student’s *T*-test, *n* = 10, *P* < 0.05) were observed, suggesting a decrease in exploratory activity, as well as enhanced levels of anxiety and aversion in SPS rats under this environment (Figure 1B). Reduced activity of SPS rats in the aversive zones indicated increased anxiety behavior rather than reduced exploration behavior.

#### Fear Conditioning in SPS Rats

There was no significant difference in the baseline level of freezing between the control and SPS rats. The SPS rats showed increased freezing in comparison with the control group in the contextural test (student’s *T*-test, *n* = 10, *p* < 0.05). However, no significant difference in the freezing level was seen between the SPS and the control rats in the auditory cued fear test (Figure 1C). In the sensibility test to the foot-shock, there was no significant difference in the minimum current which induced notice, flinch and vocalize between control and SPS groups (Figure 1D).

### Decreased LTP

**Figure 2:**
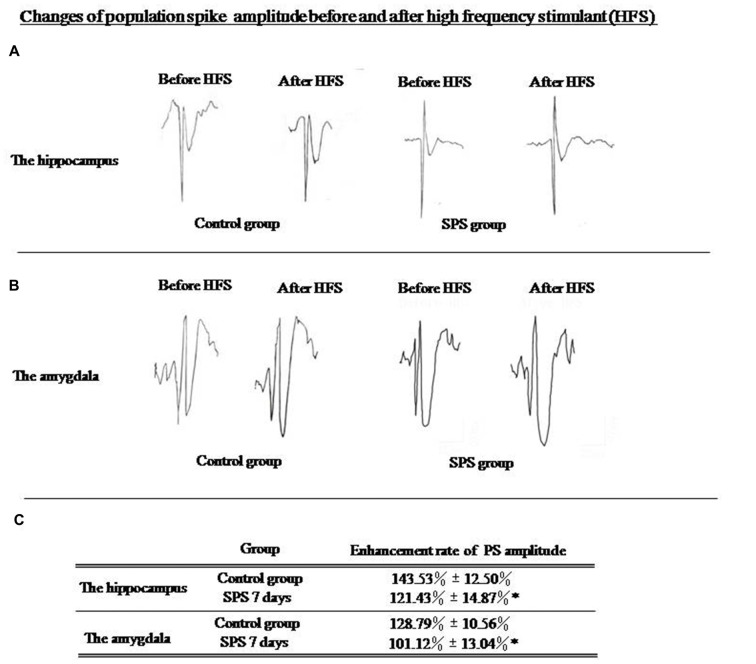
**(A)** Evoked population spike (PS) wave formed before and after high frequency stimulation (HFS) in hippocampus; **(B)** Evoked PS wave formed before and after high frequency stimulation (HFS) in amygdala. **(C)** SPS rats showed significantly decreased enhancement of evoked PS amplitude in hippocampus and amygdala compared with control rats (*P < 0.05 vs. the control group).

An increase of 143.53% ± 12.50% in the PS amplitude could be observed in the hippocampus of control rats, whereas that in SPS rats had increased by 121.43% ± 14.87% (Figure 2A). Amplitude of evoked PS in the amygdala, had increased by 128.79% ± 10.56% in the control group, while that in SPS rats had increased by only 101.12% ± 13.04% (Figure 2B). Thus, differences in enhancement of PS amplitude in both brain regions between two groups were statistically significant (Figure 2C). Decreased in LTP after SPS indicated altered plasticity in the amygdala and the hippocampus, which could be associated with formation of altered fear memory.

### Changes in Stathmin and Tubulin Expression

**Figure 3:**
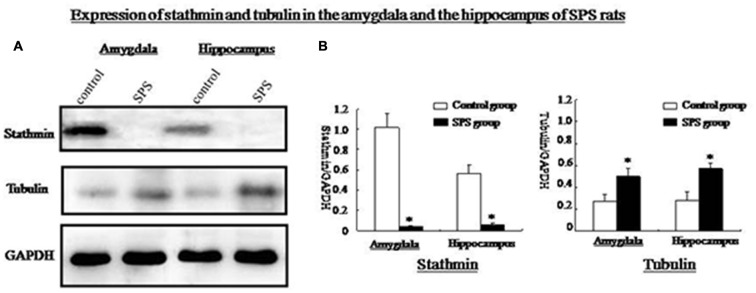
**(A)** Western blot analysis of stathmin and tubulin in amygdala and hippocampus from control and SPS groups. **(B)** Quantification of western blots showed that, stathmin was significantly decreased while tubulin was remarkably increased in both brain regions of SPS rats compared with control rats (*P < 0.05 vs. the control group).

Western blot analysis of amygdalar and hippocampal tissues showed a significant decrease in stathmin expression in SPS rats compared with the control group. Stathmin is involved in MT dynamics by regulating both the formation and disassembly of MTs. We found that tubulin expression increased significantly in both brain regions after SPS (Figure 3).

**Figure 4:**
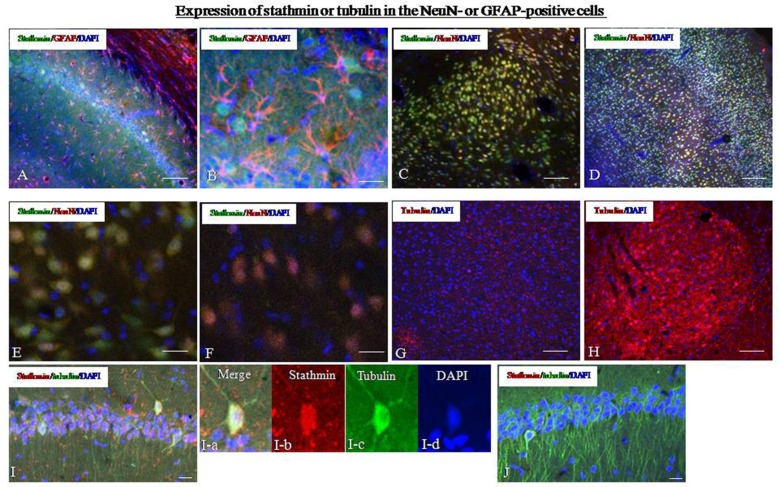
**Expression of stathmin and tubulin in the hippocampus. (A)** Dual-immunofluorescence image showing stathmin-ir and glial fibrillary acid protein (GFAP)-ir in the hippocampus of the control group. **(B)** A higher magnification image showing colocalization of stathmin-ir and GFAP-ir in the hippocampus of the control group. **(C)** Dual-immunofluorescence image showing stathmin-ir and NeuN-ir in the amygdala of the control group. **(D)** Dual-immunofluorescence image showing stathmin-ir and NeuN-ir in the cingulate cortex of the control group. **(E)** A higher magnification image showing colocalization of stathmin-ir and NeuN-ir in the amygdala of the control group. **(F)** A higher magnification image showing decreased stathmin in the amygdala of the SPS group. **(G,H)** Expression of tubulin in the amygdala of the control group **(G)** and the SPS group **(H)**. **(I,J)** Colocalization of stathmin- and tubulin-ir in the hippocampal CA1 region of control **(I)** and SPS group **(J)**. The magnification image of colocalzation of stahtmin- and tubulin-ir were showed in the **I-a** (merge), **I-b** (stathmin), **I-c** (tubulin0 and **I-d** (DAPI; *P < 0.05 vs. the control group; Bar in (**B,D–F,I,J**: 100 μm; Bar in **A,C,G,H**: 20 μm).

To determine the types of cells that express stathmin in the amygdala and hippocampus, we compared the localization of stathmin-immunoreactivity (ir) with the localization of markers of different cell types. We first examined stathmin-ir expression in glial cells by determining its colocalization with glial fibrillary acid protein (GFAP). Dual-immunofluorescence experiments showed that stathmin-ir was expressed in hippocampal glial cells in the control group (Figures 4A,B). Next, we found that stathmin-ir was present primarily in neuronal nuclear antigen (NeuN)-positive cells; NeuN is a marker of mature neurons and is expressed in principal cells and interneurons. Cells that coexpressed stathmin-ir and NeuN-ir were observed in the hippocampus (data not shown), the amygdala (Figures 4C,E), and the cingulate cortex (Figure 4D). However, no coexpression was found in the SPS animals because of a lack of stathmin -ir (Figure 4F). The intensity of Tubulin –ir increased significantly in SPS rats (Figure 4G) compared with the control group (Figure 4H), which was consistent with the western blot results. Finally, we performed dual-immunofluorescence experiments to show the colocalization of stathmin and tubulin. Stathmin- and tubulin-ir colocalized in number of cells of the hippocampal CA1 region in the control group (Figure 4I), but no coexpression cells were found after SPS stimulation because of decreased stathmin-ir (Figure 4J).

### Changes in Rin1 Expression

**Figure 5:**
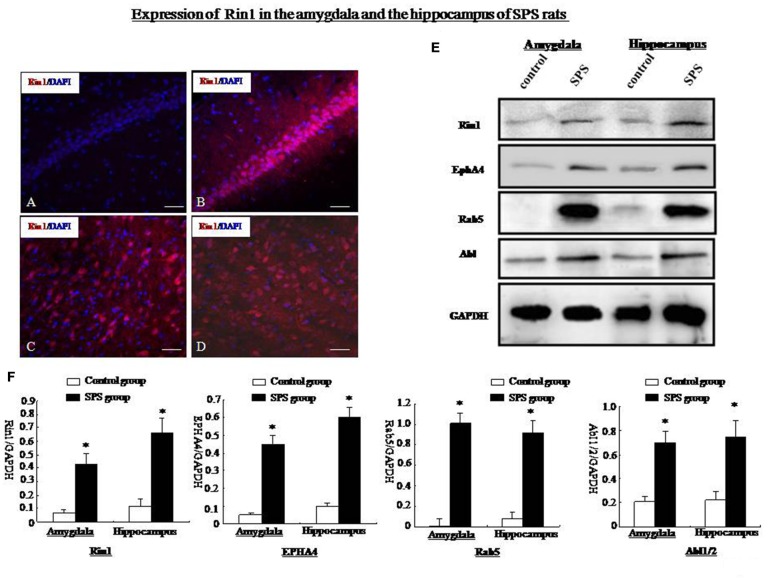
**(A)** Rin1-ir in the hippocampus of the control group. **(B)** Rin1-ir in the hippocampus of the SPS group. **(C)** Rin1-ir in the amygdala of the SPS group. **(D)** Rin1-ir in the cingulate cortex of the SPS group. **(E)** Western blots showing expression of Rin1, EphA4, Rab5, and Abl in the amygdala and hippocampus of both groups. **(F)** Quantification of western blots showing higher expression of Rin1, EphA4, Rab5, and Abl in the amygdala and hippocampus of the SPS group compared with the control group (*P < 0.05 vs. the control group; Bar: 20 μm).

The Rin1 gene encodes a Ras effector protein that signals through downstream Rab5 and Abl to positively regulate endocytosis and cytoskeletal remodeling. Therefore, we examined expression of Rin1 and its downstream effectors, EphA4, Rab5, and Abl. Rin1 levels were extremely low in control rats. Therefore, we were unable to obtain precise localization data for Rin1 protein (Figure 5A). Other studies have suggested that low Rin1-ir expression is due to a lack of antibody detection of endogenous Rin1 in brain tissue. We found high Rin1-ir expression in the hippocampus, (Figure 5B), amygdala (Figure 5C), cingulate cortex (Figure 5D), and thalamus of SPS rats (data not shown). Western blot analysis showed a significant increase in Rin1 protein in the amygdala and hippocampus of SPS animals compared with control rats (student’s *T*-test, *n* = 4, *p* < 0.05). The downstream effectors EphA4, Rab5, and Abl were also expressed at higher levels in both regions of the SPS group than in control rats (student’s *T*-test, *n* = 4, *p* < 0.05; Figures 5E,F).

**Figure 6:**
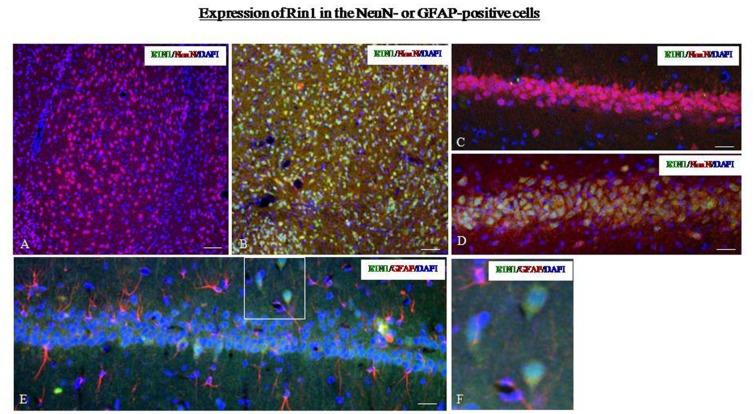
**(A,B)** Dual-immunofluorescence images for Rin1-ir and NeuN-ir in the amygdala of the control **(A)** and SPS **(B)** groups. **(C,D)** Dual-immunofluorescence images for Rin1-ir and NeuN-ir in the hippocampus of the control **(C)** and SPS **(D)** groups. **(E)** Rin1-ir in GFAP-positive cells in the hippocampus of SPS rats. **(F)** A higher magnification image of the area in the white box in panel **(E)** (Bar in **A,B**: 100 μm; Bar in **C–E**: 50 μm).

Next, we studied the localization of Rin1-ir using markers for different cell types. In SPS animals, Rin1-ir was mainly expressed in NeuN-positive cells in the amygdala (Figure 6B) and hippocampus (Figure 6D) and was not expressed in GFAP-positive cells (Figures 6E,F). In control rats, Rin1-ir was not expressed in these brain regions (Figures 6A,C).

**Figure 7:**
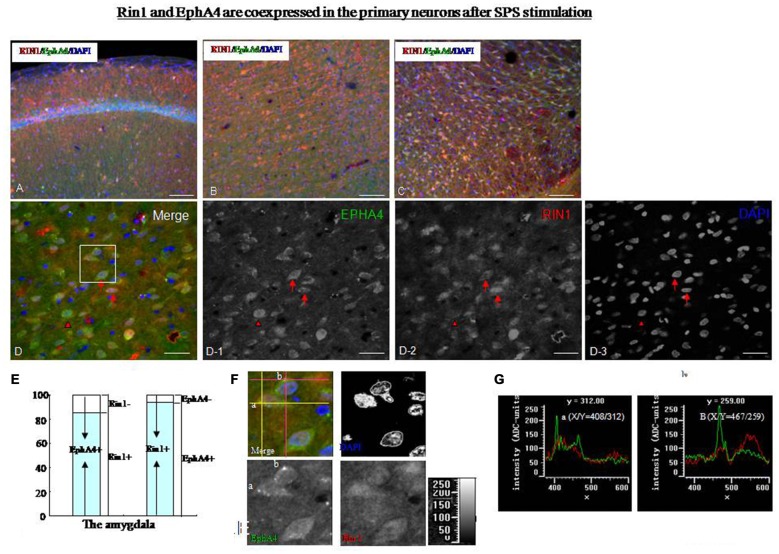
**(A–C)** Dual-immunofluorescence images showing that Rin1-ir and EphA4-ir were colocalized in the hippocampus **(A)**, cingulate cortex **(B)**, and thalamus **(C)** of SPS rats. **(D)** Higher magnification image of the amygdala shows colocalization of Rin1/EphA4 (arrow) and EphA4- or Rin1- positive cells (arrowhead). EPHA4: **D-1**; RIN1: **D-2**; DAPI: **D-3**. **(E)** Statistical analysis indicated that about 88% of EphA4-positive cells were Rin1-positive, and 93% of Rin1-positive cells were EphA4-positive in the amygdala. **(F)** Higher magnification images of the area in the white box in panel **(D)**. Some bright clusters were detected (merge). Two clusters (a and b) were selected by positioning the coordinates (a: intersection of two yellow lines; b: intersection of two pink lines). **(G)** Intensity of points a and b. The green line shows the intensity of EphA4, and the red line shows the intensity of Rin1. EphA4 was expressed at peak intensity at points a (*X*/*Y* = 408/312) and b (*X*/*Y* = 467/259; *P < 0.05 vs. the control group; Bar in **A–C**: 100 μm; Bar in **D**: 20 μm).

Rin1 and EphA4 were coexpressed in primary neurons after stimulation by SPS. Rin1 interacts with EphA4 in excitatory neurons and mediates endocytosis of EphA4. To explore the relationship between Rin1 and EphA4 under SPS conditions, we performed a dual-immunohistofluorescence assay using EphA4 and Rin1 antibodies. We found extremely low levels of Rin1- and EphA4-ir in the brains of control rats (data not shown). Therefore, we were unable to obtain precise Rin1 and EphA4 protein localization data. We found a high Rin1-/EphA4-ir coexpression ratio in the hippocampus (Figure 7A), cingulate cortex (Figure 7B), thalamus (Figure 7C), and amygdala (Figure 7D) of SPS rats. A magnified image of cells in the amygdala revealed colocalization of Rin1-/EphA4-ir (Figure 7D, arrow) and Rin1- or EphA4-positive cells (Figure 7D, arrowhead). About 88% of EphA4-positive cells were Rin1-positive, and 93% of Rin1-positive cells were EphA4-positive in the amygdala and the hippocampus of the SPS group (Figure 7E). Punctate fluorescence was detected in the cytoplasm and axons of cells in which EphA4- and Rin1-ir colocalized (Figure 7F). Cluster intensity was analyzed using single-channel pseudo-color mode, and we found that the high intensity clusters were only EphA4. We selected two clusters (a and b) and measured their intensity by positioning the coordinates (intersection of two pink lines/yellow lines). EphA4 showed peak intensity at points a and b (Figure 7G, green line), whereas Rin1 did not (red line), indicating that the frequency of the peak between EphA4 and Rin1 was inconsistent and that EphA4 immunoreactive intensity was higher at a/b than that at other points.

### Roles of Rin1 and Stathmin in Response to Immobilization (IM) and a Loud Sound

**Figure 8:**
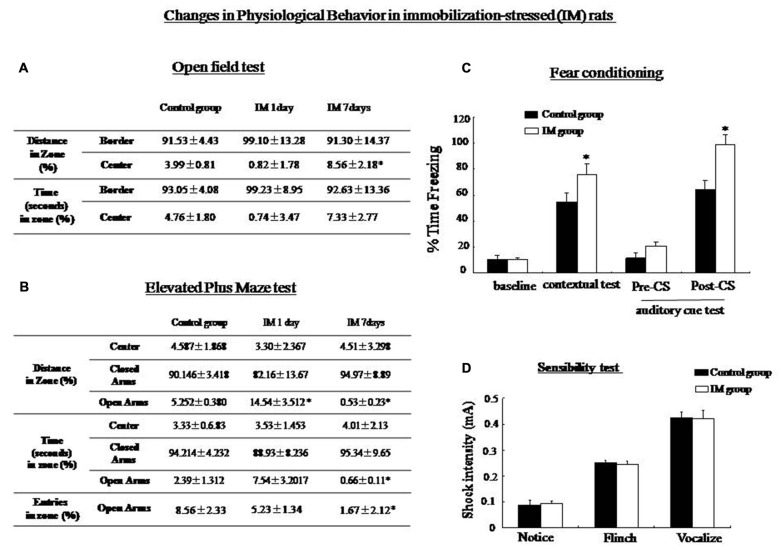
**(A)** Open-field test: The immobilization (IM)-stressed rats showed a greater percentage of distance in the border zone compared with control rats. **(B)** Elevated plus maze: IM-stressed rats showed shorter distance, less time and less entry number in the open arm compared with control rats. **(C)** Conditioning test: The percentage of time spent freezing in contextual and auditory cue fear conditioning (post-CS) were significantly higher in the IM rats than in the control rats (*P < 0.05 vs. the control group). No significant difference was observed in baseline and pre-CS of the auditory cue fear conditioning between control and IM rats. **(D)** sensibility test to the foot-shock, no significant difference in minimum current strength which induced notice, flinch and vocalize was found between control and IM rats.

SPS consisted of multiple stresses. An IM stimulus was provided to detect changes in Rin1 and stathmin expression under a non-SPS like stress condition, with an aim to explore whether changes in both genes were specific to SPS. The OF test showed a significant increase in distance from the center zone after 7 days in the IM rats compared with control rats (One-way ANOVA, *n* = 5, *p* < 0.05; Figure 8A). The EPM results revealed a significant decrease in distance, time and the number of entries into the open arms 7 days after IM exposure (One-way ANOVA, *n* = 5, *p* < 0.05; Figure 8B). Contextual and auditory cue fear conditioning test also revealed the significant increase in level of freezing in IM rats compared with control rats (One-way ANOVA, *n* = 10, *p* < 0.05; Figure 8C). In the sensibility test to the foot-shock, there was no significant difference in the minimum current which induced notice, flinch and vocalize between control and IM groups (Figure 8D).

**Figure 9:**
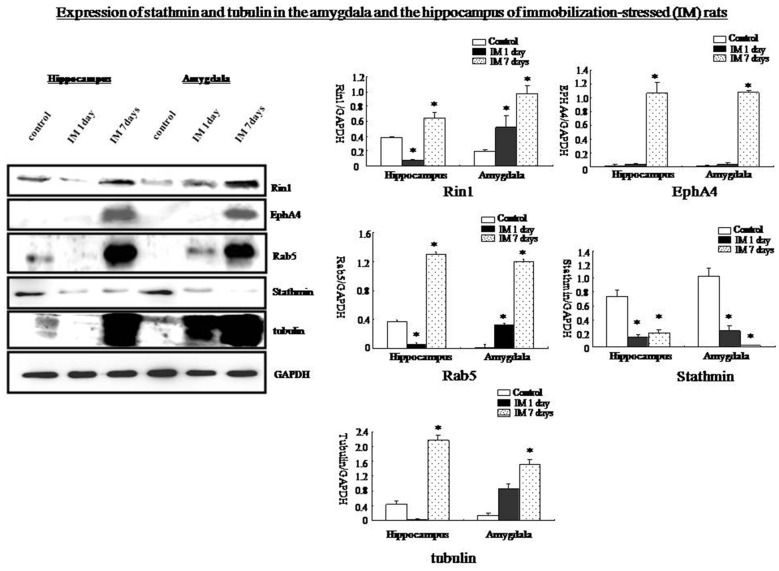
**Western blots showing that Rin1, EphA4, Abl, Rab5, and tubulin expression increased significantly in the amygdala and hippocampus 7 days after the IM-stress stimulation compared with the control rats; in contrast, stathmin expression decreased**. Quantification of western blots showing lower expression of Rin1, EphA4, Rab5, and tubulin in the amygdala and hippocampus of the IM 1 day group and higher expression in the IM 7 days group compared with the control group except stathmin (*P < 0.05 vs. the control group).

**Figure 10:**
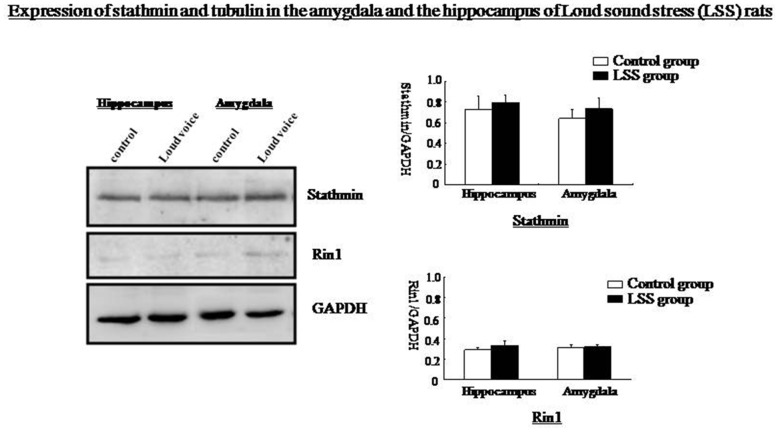
**Western blots showing no changes in stathmin or Rin1 expression after the loud sound stimulus**.

Western blotting showed that Rin1, EphA4, Abl, Rab5, and tubulin expression decreased significantly in the amygdala and the hippocampus at 1 day and increased significantly at 7 days after the IM stimulation, whereas stathmin expression decreased at 1 day and 7 days after the IM stimulation (One-way ANOVA, *n* = 4, *p* < 0.05; Figure 9). These results were consistent with those observed after SPS, despite the distinct magnitude of changes in expression. Changes in stathmin or Rin1 expression could not be observed after a loud sound stimulus (student’s *T*-test, *n* = 4, *p* < 0.05; Figure 10).

## Discussion

### Behavioral Change in SPS Rats

Three behavioral tests (namely, OF test, elevated plus maze and fear-conditioning test) are tested in the present study to examine the behavioral changes in SPS- and immobilization-stressed rats. OF and EPM were used to be measured anxiety level, exploratory activity, and aversion. EPM results showed that SPS induced decreased levels in entries number/time/distance of the open arms and increased level in the closed arms. OF test exhibited decreased locomotor activity within the inner regions of the field. These results suggested distinctly enhanced anxiety level, enhanced aversion and decreased exploratory in SPS rats in comparison with control rats, which are consistent with results from other studies on SPS and may be important for understanding the human condition of PTSD. Our fear-conditioning tests showed higher freezing level in SPS rats than in control group in contextual memory, but not for auditory cue memory. It is well known that the amygdala has an important role in auditory-cued and contextual fear conditioning. In auditory-cue conditioning, direct projections from the thalamus and/or from the auditory cortex to the lateral amygdala (LA) are thought to be critical (Romanski and LeDoux, 1992; Li et al., 1996; LeDoux and Muller, 1997). Thus, lesions of the LA, but not of the basolateral amygdala (BLA), accessory basal, or medial nucleus of the amygdala can block auditory-cue conditioning (Nader et al., 2001). Therefore, the different behavioral and electrophysiological outcomes obtained in contextual and auditory-cued fear conditioning may be due to a different expression in Rin1 and Stathmin in subnuclei of the amygdala after SPS stimulation. Studies from Toledano and Gisquet-Verrier (2014) found that SPS rats showed decreased or unchanged level of acoustic startle response, which is consistent with our results. Studies from Imanaka et al. (2006) also reveals markly elevated freezing level in acquisition of fear conditioning in SPS rats compared with that in control rats. But studies from Knox et al. (2012) shows no effect of SPS on the acquisition of fear conditioning. It has been reported that the hippocampus and the amygdala are involved in contextual conditioning and encode memories in multiple cues is associated with the aversive event (Phillips and LeDoux, 1994; Calandreau et al., 2005). During traumatic process, dysfunction in both regions can bias the formation of multiple cues and exaggerate fear responses under multiple environments (or contexts; Achesona et al., 2012). Beyond that, we also found similar behavioral changes in immobilization-stressed rats. IM rats showed an enhanced contextual memory and auditory cued memory in comparison with control rats, which is different from what was observed in the SPS rats; this suggests that SPS and other traumatic stressors could induce different changes in different cued fear conditioning.

### Dysfunction in Stathmin after SPS Exposure

Our results suggest a loss of stathmin expression after SPS exposure. It is first found that stathmin is highly expressed in most brain regions, such as the hippocampus and the amygdala, which are the main regions regulating emotional memory. Stathmin knockout mice exhibited normal neuronal morphology, decreased memory, and recognizeably reduced less danger in innately aversive situations, suggesting that the loss of stathmin expression impacts innate and learning anxiety-related behavior (Shumyatsky et al., 2002). Our OF test results showed that SPS rats spent less time in the center region, which is in contrasts with results from the knockout mice. These opposite results may be attributed to different manifestations of a basic-fear disorder or the comprehensive effects of changes on expression of multiple genes in SPS animals. Stathmin is probably regulated by a basic fear because it is highly expressed under normal conditions. Studies from knockout animals found reduced contextual fear memory and impaired dentate gyrus LTP in stathmin−/− mice, indicating that stathmin was a positive regulator of fear memory (Shumyatsky et al., 2002; Uchida et al., 2014; Zhang et al., 2015). In addition, abilities to properly assess a threat, provide parental care, and interact socially as adults are deficient in stathmin knockout animals (Martel et al., 2008). Stathmin plays a negative role in regulating MT formation. A lack of stathmin and increased tubulin expression may be indicative of greater MT formation and decreased MT dynamics in SPS rats. MTs may be important for synaptic activity and cellular transport in the case of transporting important molecules and organelles to the synapse (Westermann and Weber, 2003). Enhanced MT function may be required to maintain basic physiological functions of cells under the condition with SPS-induced high apoptosis ratio condition (Hirokawa and Takemura, 2005). Changes in cytoskeletal proteins integrin, vinculin and connexin 43 are also found in our previous study (Li et al., 2015), which are consistent with increased MTs. Few studies at present have focused directly on stathmin expression in fear-related psychological disorders. A recent study from Uchida showed deficit of stathmin reduced contextual fear memory and impaired dentate gyrus LTP (Uchida et al., 2014). As is reported in one clinical study, stathmin expression is associated with re-experiencing of PTSD symptoms (Cao et al., 2013). Finally, an opposite change in stathmin expression is found in one study; besides, blast-related traumatic brain injury can increase stathmin expression in amygdala as well as anxiety levels (Elder et al., 2012).

### Rin1 Functions after Stimulation by SPS

Rin1 is expressed postnatally in the brain, which is dramatically reduced in expression in adult brains (Bliss et al., 2010). Here, we showed increased expression of Rin1 and its downstream effectors Rab5 and Abl in the hippocampus and amygdala of SPS rats. Our behavioral tests showed an enhancement of freezing time in SPS rats compared with the control group in the contextual fear conditioning, not auditory fear. It is discovered in our previous study that spatial-dependent memory in SPS rats is deficient as well. Rin1−/− mice display enhanced auditory fear conditioning through increasing fear acquisition/retention with deficits in extinction (Dhaka et al., 2003; Bliss et al., 2010). As is reported by Bliss, Rin1−/− mice had reduced latent inhibition, indicating that Rin1 has a limited effect on establishing memories (Bliss et al., 2010). Few existing studies that investigated the relationship between Rin1 and fear have been limited to Rin1 knockout animals. Direct evidence showing expression and function of Rin1 in a fear-related psychological disorder is lacking at present. Increased Rin1 level in hippocampus and amygdala of SPS rats is found in this research, suggesting a possibility of altered amygdala-hippocampal interactions. However, according to the present data, no direct evidence is available to conclude that increased Rin1 expression can inhibits fear memory in SPS rats. Instead, it can only be achieved by interventional studies in amygdala and hippocampus. Abl is reported to affect short-term synaptic plasticity (Moresco et al., 2003). Rab5 controls endocytosis of cell surface receptors such as AMPA (Bliss et al., 2010). It has been also reported that stathmin mutations disrupt GluA2 (one subunit of AMPA) localization (Uchida et al., 2014), suggesting AMPA could be a common target of stathmin and Rin1. It would be helpful for understanding functional link between both independent molecules in stress disorder. Rin1 coordinates stimulation of the Abl and Rab5 signaling pathway by integrating actin remodeling, recycling receptors by endocytosis, and trafficking at the postsynaptic membrane. Our dual-immunofluorescence assay revealed that Rin1 was expressed in primary neurons. Rin1 and EphA4 coexpression in primary neurons of SPS rats suggests that Rin1 interacts with EphA4 while regulating endocytosis, which is relevant to neuronal plasticity (Deininger et al., 2008). Interactions between Rin1 and EphA4 have been examined in several cell lines. Increased Rin1 expression inhibited enhancement of LTP in the amygdala by suppressing EphA4 internalization and function in SPS rats. High intensity EphA4-stained clusters were localized on the surface of primary cells, which is an area of internalization.

Taken together, our data strongly suggest that the change in stathmin and RIN1 can be observed in the hippocampus and the amgydala in SPS rats. However, behavioral expression of knockout animals with both genes suggests that increased RIN1 and decreased stathmin should inhibit fear memory formation, which contradicts our behavioral test results (increased contextual freezing and unchanged auditory freezing). Two possibilities that may explain the inconsistencies between the observed behaviors and expression of the genes: first, the interaction of RIN1 with stathmin may induce different expression of the individual knockout gene since they share a common target, which is AMPA in the pathways of stathmin and Rin1. Second, such inconsistency may derive from the comprehensive effect of changes in expression of multiple genes in SPS animals.

### Stathmin and Rin1 Expression after Other Traumatic Stressors

SPS represents a very specialized stress, but whether other traumatic stressors can change stathmin and Rin1 expression remains unknown. Two additional stressors, IM stress and LSS, are used in this research. Expression of Rin1, EphA4, Abl Rab5 and tubulin in amygdala and hippocampus is distinctly increased after IM stimulation, whereas stathmin expression is decreased, which is consistent with SPS results. Behavioral tests indicate abnormal innate fear and enhanced fear, demonstrating that not only SPS but also other traumatic stressors can change expression of stathmin and Rin1. Thus, changes in both genes may not be specific to PTSD-like stress; instead, they may be specific to a broader spectrum of traumatic stresses. The lack of changes in stathmin and Rin1 expression after a LSS is consistent with our hypothesis. However, changes in stathmin and Rin1 expression should be further examined in additional trauma-related psychological disorders.

### Unexpected Finding and the Limitation

It is shown in the present study that SPS induces increased contextual fear conditioning, increased Rin1 level while decreased Stathmin level in hippocampus and amygdala. However, Rin1 knockout mice demonstrate enhanced fear conditioning, while Stathmin knockout mice display decreased fear memory. The inconsistency between context conditioning and expression of Rin1/Stathmin may be explained by the comprehensive effects of changes on expression of multiple genes in SPS animals. On the other hand, Stathmin is likely regulated by a basic fear, which may be different from Rin1 in regulating fear conditioning. Therefore, further studies will be required to explicitly address the effects of Rin1/Stathmin on fear conditioning in SPS model.

Our study does not illustrate a direct involvement of Stathmin or Rin1 in the enhanced fear conditioning observed in rats subject to SPS or to immobilization. Therefore, interventional studies should be enrolled in future study. Such as it is, our results may provide new insight into the molecular mechanism of abnormal fear memory after exposure to trauma and a better understanding towards individual variations in PTSD susceptibility and therapy.

## Conclusion

Our data show that changes in stathmin and RIN1 in hippocampus and amgydala can be seen under the conditions of SPS and IM, suggesting that the changes in the expression of Rin1 and stathmin genes may be involved in SPS and immobilization stress. But changes in the expression of both genes may not be specific to PTSD-like stress.

## Author Contributions

FH and YS contributed to experimental design; FH, JJ, JD and HL contributed to acquisition of data; FH, BX and HL contributed to analysis of experimental data.

## Funding

This work was supported by two grants from the China National Natural Science Foundation (No. 81571324, 31200772) and Education Department of LiaoNing Province, China (No. LJQ2014083).

## Conflict of Interest Statement

The authors declare that the research was conducted in the absence of any commercial or financial relationships that could be construed as a potential conflict of interest.
